# Scenario- and discussion-based approach for teaching preclinical medical students the socio-philosophical aspects of psychiatry

**DOI:** 10.1186/s13010-023-00146-4

**Published:** 2023-11-10

**Authors:** Ya-Ping Lin, Chun-Hao Liu, Yu-Ting Chen, Uen Shuen Li

**Affiliations:** 1 Department of Medical Humanities and Education, School of Medicine, National Yang Ming Chiao Tung University, Taipei City, 112 Taiwan; 2 Institute of Public Health, National Yang Ming Chiao Tung University, Taipei City, Taiwan; 3 Department of Child and Adolescent Psychiatry, Chang Gung Memorial Hospital, Linkou Taoyuan City, Taiwan; 4 School of Medicine, National Yang Ming Chiao Tung University, Taipei City, Taiwan

**Keywords:** Medical education, Philosophy, Psychiatry, Scenario-based approach, Social structure

## Abstract

**Background:**

This study used a scenario- and discussion-based approach to teach preclinical medical students the socio-philosophical aspects of psychiatry and qualitatively evaluated the learning outcomes in a medical humanities course in Taiwan.

**Methods:**

The seminar session focused on three hypothetical psychiatry cases. Students discussed the cases in groups and were guided by facilitators from multiple disciplines and professions. At the end of the semester, students submitted a narrative report comprising their reflections on the cases and discussions. The authors utilized content analysis to categorize students’ narratives into three facets, namely, the philosophical, social and individual.

**Results:**

In total 163 preclinical medical students participated in the class; 150 of them mentioned the scenario-based lesson in their reports; 33.3% of these reports discussed the case at the philosophical dimension (*n* = 50), 45.3% at the social dimension (*n* = 68), and 26.6% at the individual dimension (*n* = 40). Four major themes emerged: (1) a psychiatric diagnosis has far-reaching consequences for an individual’s life, (2) the social structure affects how patients experience psychiatric disorders, (3) students related personal experience or those of friends and family to understand psychiatric disorders, and (4) medical humanities are of particular importance in psychiatric education.

**Conclusions:**

This study demonstrated that the scenario-based discussions led by a multidisciplinary team of facilitators can benefit medical students with limited clinical experience to contemplate the socio-philosophical aspects of psychiatry. The authors suggest that this pedagogical model during preclinical education should be encouraged.

## Background

In 1928, Adolph Meyer remarked on the complexity of psychiatry when he wrote, “I sometimes feel that Einstein, concerned with the relativity in astronomy, has to deal with very simple facts as compared to the complex and erratic and multicontingent performances … which we psychiatrists are concerned with” [1]. Because of the complexity of the brain, the mind, and human behavior, psychiatry involves the interface of biological, psychological, and environmental factors [2]. In clinical practice, various perspectives through which a patient can be seen are integrated into a “biopsychosocial model” [3]. Social determinants, such as adverse childhood events, racism, and poverty, play major roles in mental health [4]. Psychiatric residency education thus has begun to integrate psychosocial aspects of the discipline, such as sociological and anthropological perspectives, into the biomedical model to help residents understand how social adversity affects those with psychiatric disorders [5].

Over the past century, psychiatry has undergone a slow but substantial paradigm shift from focusing on the anthropological and philosophical basis toward the biomedical basis of neuropsychiatric disorders [6, 7]. To help students understand the complex nature of psychiatry, certain thinking tools borrowed from the field of philosophy might be useful. Philosophy and psychiatry have some common features: both examine reality, perception, thought, affect, and free will [6]. Certain philosophical thinking skills, such as reflecting, conceptual thinking, and questioning, are fading from the daily practice of biomedical psychiatry and psychiatric education [6].

Most studies on ethics in psychiatric education have focused on John Gregory’s (1724–1773) framework of professional virtues, namely integrity, compassion, self-effacement, and self-sacrifice [8, 9]. Although some attempts have been made to integrate philosophy into psychiatric residency training, only a few studies have addressed the integration of philosophy into psychiatric training in medical school. The teaching of psychiatry in Taiwanese medical schools often emphasizes the biomedical over the social-philosophical aspects. Also, the two aspects are taught in a dichotomized manner (e.g., psychopathology or psychopharmacology vs. medical humanities). This raises the question of how to teach medical students the socio-philosophical aspects of psychiatry, even before they had clinical experience.

In this study, we implemented a scenario- and discussion-based teaching model to introduce preclinical medical students to the socio-philosophical aspects of psychiatry. At the end of the course, we reviewed students’ narrative reports to evaluate whether the course has broadened their understanding of these topics. In this paper, we describe the design of the course with Taiwanese culture–based scenarios, and we analyze how students have deepened their understanding of the socio-philosophical aspect through discussions.

## Methods

### Course design

In Taiwan, medical education is carried out through a six-year undergraduate program, followed by residency training. Medical ethics is generally either taught in a single course taken during the first 2 years of medical school or is assimilated at bedside during residency training and clinical practice. These courses are often developed and taught solely by clinical professionals. In 2013, the lead author (YPL), a philosophy professor with years of experience in teaching medical ethics, designed a new longitudinal integrated ethics curriculum in the Medical School of Chang Gung University. The course, titled “Medical Humanities and Clinical Ethics” is a compulsory 2-credit weekly (2 hours each week for 18 weeks in a semester) course for the fourth-year preclinical medical students. The first 2 weeks of the course were introductory sessions on medical ethics, followed by 4 seminar sessions, each was 2 hours per week and lasting for 2 weeks, for the group discussion of clinical cases among students. The discussions were facilitated by teaching members from multiple disciplines and professions, including ethics, law, sociology, anthropology, and medicine. The multidisciplinary team took advantage of their diverse background, adding different dimensions to the discussion. Students were inspired to have a comprehensive understanding of the social implications of the psychiatric diagnosis and treatment, as well as the cultural context where patients are embedded in.

This study was conducted in the 2020 fall semester, centering around one of the seminar sessions on the socio-philosophical aspects of psychiatry. During the seminar session, we first delivered a lecture on psychiatric diagnosis and the Diagnostic and Statistical Manual of Mental Disorders (DSM) system, after which we provided three scenarios for students to discuss. The scenarios provided are commonly encountered in daily Taiwanese psychiatric practice; socio-philosophical issues are incorporated into the scenarios to guide student’s discussion on those issues. Essential diagnostic criteria were provided to the students before the discussion and the clinical diagnosis of each case was stated in the scenario.

#### Case 1

A 35-year-old woman, Ah-Yu, had a history of postpartum depression and bipolar disorder. She was a full-time homemaker after pregnancy according to the conventional division of labor in Asian societies. Before that, she was a manager in the finance industry and had regular arguments with her husband over financial issues.

Two months ago, Ah-Yu began to present manic symptoms, including heightened goal-directed activity (attending several economic conferences), hypertalkativity, frequent shopping sprees, and financially risky behavior (increased investments). Her husband sent her to a psychiatric ward where she was diagnosed with bipolar disorder. He also asked for adult guardianship of his wife based on her limited capacity.

#### Case 2

A 16-year-old girl, Hsiao-Yen, was sent to the pediatric clinic after experiencing dizziness. The pediatrician noted that she was 160 cm tall but weighed only 32 kg; moreover, she had hypothermia and secondary menopause. Therefore, she was transferred for psychiatric evaluation.

During psychiatric evaluation, Hsiao-Yen met the diagnostic criteria for anorexia nervosa. The psychiatrist also found that she had an experience of being bullied because of her figure. She was introverted, nervous, and self-demanding. Her mother was a professor and her father was a senior manager. Her family did not seem to be very close. The psychiatrist recommended admitting the patient to the psychiatric ward. After being told the reason for her admission, the patient refused to be admitted.

#### Case 3

A boy, Hsiao-Ming, aged 7 years and 6 months was taken to the psychiatric outpatient department because his elementary school teacher reported that he was “talkative, fidgeted often, inattentive, and had frequent conflicts with his classmates” in school. After taking a detailed history, the psychiatrist attributed his clinical functional impairment to attention deficit hyperactivity disorder (ADHD). Hsiao-Ming’s single mother reported that his adjustment in kindergarten was fair, which may have been due to the patience of his kindergarten teacher. The Conners Continuous Performance Test revealed a borderline result, but the SNAP-IV questionnaire from the elementary school teacher and his mother indicated that he was over the diagnostic cut-off point. The psychiatrist discussed pharmacological and non-pharmacological treatment plans with the mother. As a single mother with a low-paying job, she could not afford behavioral therapy, and she was too burnt-out to improve her parenting skills. She preferred pharmacotherapy.

#### Group discussion

Students were randomly divided into groups of 10 to 12. Each group was assigned one of the scenarios. A facilitator was assigned to each group to lead the discussions. The facilitators were either clinical professionals or medical humanities professors, and all had cross-specialty teaching or research experience. Among the teaching team, only one was a psychiatrist. The students were asked to discuss the psychosocial aspects of the case and identify the challenges in these clinical scenarios. As students’ understanding of the clinical cases might be limited by their own experiences, the input from the multidisciplinary facilitators was a key to enrich students’ imagination of the everyday clinical environment, enabling them to have a thorough and critical discussion in the process.

#### Data collection

At the end of the semester, students were asked to submit narrative reports on their personal reflection on the cases, what they have learnt from the discussion, as well as their feedback to the course design. There was no requirement of the format or length of the report. The authors (YPL and YTC) collected the narratives and anonymized them before analysis.

### Data analysis

We used a cross-sectional study design and applied two different coding schemes to analyze the reports [10]. First, we defined three facets of discussion topics [11]. Facet A, the philosophical facet, was defined as discussion focused on the conceptual aspects of the following issues: the labeling effect of psychiatric disorder, the differentiation of the normal and the pathological, and the competing nature between categorical and dimensional thinking [7]. Facet B, the social facet, was defined as discussion focused on patients’ social environments, including their families, living situations, and the social structure [12]. Facet C, the individual facet, was defined as discussion focused on how the patient coped with the psychiatric phenomenon at the individual facet, including their reception of pharmacotherapy, psychotherapy, or educational resources. The three facets were not considered to be hierarchical, although our course aimed to focus on Facets A and B (socio-philosophical aspects), whereas Facet C was much closer to the clinical thinking process. The narratives could be labeled with more than one facet.

Second, we analyzed the narratives and coded the ideas students had expressed in the narratives using qualitative content analysis. Qualitative content analysis is conducted to explore the meaning of recorded communication and is commonly used in multimethod studies [13], including in medical education [11, 14, 15]. In this study, content analysis provided a deep understanding of how medical students construct their views of the socio-philosophical aspects of psychiatry.

The authors (CHL, YPL and YTC) immersed themselves in the narratives by reading and rereading as they independently coded the reports before cross-checked their analysis. Disagreements were found in 15% of the codes; those were later discussed and resolved by reaching consensus. Content analysis focused on what the students learned and how they understood the socio-philosophical aspects of psychiatry during the course. The narratives were coded by its focus of description. Related codes were categorized into major themes. Among the major themes, thick descriptions that reflected students’ subjective experience were selected for presentation in this paper.

## Results

Among a total of 163 preclinical medical students in the class, 3 students failed to turn in the final narrative report before the deadline and 10 did not mention any content from the scenario-based lesson. Of the 150 students who mentioned the scenario-based lesson, 50 (33.3%) discussed Facet A topics (e.g. stigma and the influences of psychiatric diagnosis), 68 (45.3%) discussed Facet B topics (e.g., gender inequality and social structures such as school or family), and 40 (26.6%) discussed Facet C topics (individual management of psychiatric problems). This finding shows that a relatively high proportion of preclinical medical students were able to contemplate the philosophical and social aspect of psychiatry before they had any clinical experience.

Four major themes emerged in the narrative reports: (1) a psychiatric diagnosis has far-reaching consequences for an individual's life, (2) the social structure affects how patients experience psychiatric disorders, (3) students related personal experience or those of friends and family to understand psychiatric disorders, and (4) medical humanities are of particular importance in psychiatric education.

### A psychiatric diagnosis has far-reaching consequences for an individual's life

Making a diagnosis is a critical part of medical treatment, but it also has the risk of stigmatizing and labeling patients as “insane” or “abnormal.” Many students were able to point out that psychiatric diagnoses have an impact beyond their medical function, on the psychosocial aspects of the patients.

One of the students stated,

“Diagnosis is a double-edged sword. It makes communication between medical professionals convenient and allows doctors to prescribe precisely. Diagnosis also entitles patients to necessary resources. However, diagnosis also brings labeling and stigmatization and causes patients who have not reached the clinical [diagnostic] threshold to remain untreated. In the field of psychiatry, concerns about labeling and stigmatization are far more serious than they are for most diseases in other fields.” (#125)

While psychiatric diagnosis may enable patients to get access to resources, it may also cause adverse effects. Noticing the influence of psychiatric diagnosis, students began to reflect on the meaning of making a psychiatric diagnosis.

“I always thought that doctors can proactively look for resources to bring welfare to patients. However, when we talked about the issue of mental illness, [we observed that] a rushed diagnosis and prescription may cause labeling because people with mental illness are still stigmatized by society. Once they are diagnosed with mental illness, their choices of career and education will be limited; their right of residence will be affected too.” (#148)

We found that students were able to weave psychiatric disorders into a wider social context instead of simply regarding them as medical issues. With this awareness of the interaction between psychiatric disorders and society, students began to pay greater attention to a broader social structure.

### The social structure affects how patients experience psychiatric disorders

Psychiatric disorders cannot be discussed without considering the social context. Gender, education, socioeconomic status, family structure, and power dynamics all play central roles in patients’ experiences of psychiatric disorders.

One medical student mentioned this aspect of psychiatric disorders, commenting on their group’s discussion of Case 1 (bipolar disorder).

“I think there are many issues that are outside the scope of medicine in this case. Our group discussed the case in three dimensions: law, family power structure, and labeling… We discussed the second dimension, family power structure disparities. From the description of the case, it is obvious that Ah-Yu had postpartum depression but no one cared about her physical or psychological condition. It is thought that women should stay home and take care of the children while men go out and work. As [the husband] needed to support the family with a single paycheck, financial difficulty [likely] further exacerbated their relationship.” (#22)

Commenting on the same case, another student attempted to empathize with the patient and understand the patient’s experience of bipolar disorder, viewing it as “difficulties and struggles,” as well as “resistance behaviors to the environment,” instead of illness.

“When we look at the case again without looking at the diagnosis in the last paragraph, we can actually notice that there are reasons behind the protagonist’s behavior. The reasons could be yearnings for the workplace, desires to prove herself, or attempts to resist her plight and stress. In short, it seemed more like a stressful situation commonly observed in psychological counseling.

When people behave in a seemingly abnormal way, they have nothing but an intention to be noticed by others; they want people to recognize that they are in a difficult situation. However, because of the diagnosis of bipolar disorder or other mental illnesses, it seems that the stresses and plights that make them this way have been neglected. We attribute everything to ‘having mental illness’ as if the best assistance for them is only to treat their ‘mental illness.’ In this way, we have neglected the value of those resistance behaviors.” (#84)

Many students noticed that a mere focus on the personal facet of psychiatric disorders raises the risk of ignoring the impact of the broader social structure. With the awareness of the patient’s social context and environment, some medical students demonstrated a deeper understanding of the patient’s experience, instead of merely diagnosing the disorder.

### Understanding psychiatric disorders by relating them to personal experience or the experiences of the people around them

It might be challenging for preclinical medical students with limited clinical knowledge and experience of psychiatric disorders to understand the subjective experience of patients with psychiatric disorders. Some students attempt to understand such foreign experiences by relating that to their personal experience or recalling stories of their friends and family.

One medical student related Case 3 (ADHD) to his own experience.

“When I was reading the case, it felt like I was reading a perfect description of my younger self. I remember being too active when I was young. I always talked to friends around me in class and rushed out of the classroom to play ball once the bell rang, even if the teacher had not announced that the class was dismissed... So I was particularly excited when I saw this case.” (#75)

Another student empathized with the marginalized individuals through his junior high schoolmate’s experience.

“This reminds me of a classmate from junior high school, a classmate whose ‘sexual orientation’ was a bit special... As time went by, over the 3-year course of junior high school, he was bullied by classmates who were in their rebellious stage. Very few friends supported him... I saw him change from being an extrovert to being gloomy and depressed. His performance in school and social networks got worse and worse. I was immature at the time and did not give him any help. Because of his special identity, I dared not get close to him. A few years later, I failed the medical school entrance exam and had a difficult time. It was rather suffocating and depressing. And it was during that period that I suddenly recalled this classmate from junior high school. I realized that I could understand a bit of what he might have experienced. The sense of helplessness really leaves you in despair, and he was merely a junior high school student.” (#96)

Furthermore, those personal experiences, shared with students during group discussion, were able to inspire other students who might have less relatable experience in life to understand the difficulties faced by psychiatric patients.

As a medical student shared his experience of hearing other people's stories. “One of the most striking points raised in the discussion is the lesson that a diagnosis has a great impact on the child, their future, and how other people perceive them. A member of our group shared his personal experience, saying that his younger brother had once shown ADHD-like symptoms. At that time, a teacher at his brother’s school invited a doctor to the school to make a diagnosis for his brother. Ultimately, the doctor concluded that his brother had absolutely no problem at all and was actually a genius. The diagnosis affected his brother’s future. His brother was no longer perceived as an ADHD kid... Had the doctor diagnosed him as having ADHD, his brother would have been labeled as an ADHD kid, which would have affected his whole life. ” (#127)

By sharing a personal experience, the group member made the figure of a “psychiatric patient” to be clear and solid rather than vague and imaginary. Real stories shared by the people around them, compared to the clinical scenarios for discussion, made a deeper impression on medical students. Through the discussion of such true stories, many students were able to understand the socio-philosophical aspect of psychiatry more thoroughly.

### Medical humanities are of particular importance in psychiatric education

Many students mentioned the uniqueness of psychiatry and thus the importance of medical humanities in forging students’ sensitivity in the field. The biomedical model in psychiatry is often built on quantified results and rigid criteria. It will likely eliminate the variance among different cases and ignore the tight connection between the diagnosis and the sociocultural context, including the power structure, ideologies and value judgements in the society.

“It is very different to explore psychiatric cases in comparison to other specialties. Here exists a large gray area of diagnosis, and physicians cannot recklessly attach the label of psychiatric disorders to patients who came seeking help. What we have been learning in school mostly is knowledge with model answers. Say as we examine a microscope slide in a pathology lab, we can depend on the morphology of the cells to diagnose. However, I realize that psychiatry stresses on the complexity of human-being; personalities that spread across a spectrum, those are hard to be defined as any single pathological label. To me, this kind of thinking logic is very innovative. When our group presented the case report, we were criticized by physicians and teachers for our recklessness in making a diagnosis. That was impressive for me, making me realize that what we are facing in the future is not just disease but human beings. The complexity of human beings cannot be categorized easily. What is Normal? What is Abnormal? There may only be a fine line between those two. Therefore we need to be extra careful as we diagnose and treat. We should not rush to conclusion but instead, stay longer in the gray area, adapt to the anxiety of being in the ambiguous zone and learn how to accept every possibility with an open mind.” (#111)

Many students, through the discussion of cases provided, were able to understand the sociocultural context and ideologies contained by the psychiatric diagnosis. It is hard to distinguish normality and abnormality with a single diagnostic criterion. Students gradually recognise the pervasive power entitled to physicians within physician–patient relationships and the social structure. With that in mind, they understand the importance for a good physician, or themselves as physicians-to-be, to act with humanistic literacy.

“Physicians need to stay alert all the time; they should not be dogmatic about every patient they contact and they should not have a unified way of treating patients. To make a personalized treatment plan for every single patient is a very different category from other clinical work; psychiatry has a side that deals with humanities a lot. It wanders between personal psychological state and external environmental stress, between pathophysiology and the imbalance of coordinating self identities, between illness insight and others’ (including physicians’) gaze. It is essential to dig deep into the everyday life of every single patient and try to correct or repair those emptiness, discrepancy and imbalance, with both medical and non-medical means.” (#18)

Regarding humanistic literacy in psychiatry, students mentioned empathy, care, company, being flexible, being observant, patience, open-mindedness, inclusiveness and embracing diversity. This indicates students have a good understanding of medical humanities and how they think they should act in future interaction with patients.

“Details of analytical and thinking directions have been discussed in the lesson, but what I think the most important is care, care and care. Not necessarily to sympathize but to empathize. In our life everyone must come across some puzzling crossing roads, whether or not the person involved is rational or if the grand environment is correct, what is of greatest importance is that that person should be properly taken care of and accompanied until he/she feels better.” (#96)

“It is because psychiatric illnesses are always affected by abstract emotions, and problems beyond the lesion are the ones that are most difficult to cure. Just like in this case, it is hard for a physician to intervene in the patient’s family, or to help the patient clear the roots of their problems. What we can only do beside prescribing medication is to be observant, detecting problems beyond the lesion, and through the art of communication, to help the patient move on. This kind of ability is built on soft skills outside of knowledge. Apart from being insightful on the patient’s conditions, helping the patient to get emotional reliefs through patient and heartwarming physician-patient communication is an essential quality of a good physician. Among those, the core value of empathy is a highlight of medical humanities.” (#61)

## Discussion

Medical professionalism is one of the six core competencies listed by the Accreditation Council for Graduate Medical Education and one of the key elements of this ability is to understand and respect patients’ difficulties and well-being in different social situations [16]. However, merely preaching professionalism and ethics in class might lead to a shallow understanding of the concepts and dogmatic adoption of codes of conduct. Of greater importance is students’ ability to understand others in a diverse society and act professionally. Previous studies have suggested that professionalism could be taught through situated learning with the use of literature and stories [17], and better understood by utilizing clinician-led small group discussion [18]. In this study, we implemented a scenario- and discussion-based teaching approach to stimulate preclinical medical students’ thinking and provide a foundation for the development of medical professionalism.

An earlier Taiwanese study reported that students had responded more favorably to problem-based learning led by tutors with a nonmedical background rather than by physicians [19]. We employed a teaching group of multidisciplinary background to lead the students’ discussion. Besides one psychiatrist and one mental health nurse, the course in the present study invited facilitators from a variety of fields including ethics, sociology, anthropology, law, and nonpsychiatric clinical medical specialties. It is observed in the students’ narrative reports that apart from the socio-philosophical aspect, some students were able to extend the discussion to the legal aspects of psychiatric cases, such as the psychiatric patient’s legal and mental capacity to contract. This indicates that a teaching group of multidisciplinary background could enrich the discussion with input from different perspectives.

We designed the scenarios in this study based on the quintessential psychiatric clinical cases in Taiwan, and these scenarios captured some of the characteristics in Taiwanese socio-cultural context. Students were able to identify these characteristics in their reports. In Case 1, students pointed out the rigid gender stereotypes and the patient’s vulnerability to lose autonomy under Taiwanese legal system. In Case 2, students discussed how peer pressure and an internalized ideal body image shaped the patients’ clinical expression. Furthermore, students touched on the difficulty to balance protecting the patient’s autonomy and giving timely treatment. In Case 3, students empathized with the burnout parents whose kid’s treatment options were limited, as parents are too occupied to make ends meet.

In this study, personal experiences shared in the group left a strong impression in many students’ minds. Compared with abstract psychiatric terms, students find it easier to understand the complexity of psychiatry through the vivid narratives of their everyday life experience, where students found it easier to grasp the sociocultural aspect of psychiatry. We also found that medical students often use their daily life experience to understand others’ plights. In group discussion, experiences shared by other student members of the discussion group often appeared in students’ narratives. This was particularly obvious in Case 3; many students recalled how they or their friends were diagnosed with or suspected of having ADHD. Although students might not have met psychiatric patients, they could empathize with the originally distant patients’ experience by stepping into the shoes of their peers. These similar experiences became a bridge for medical students to understand the clinical cases and the social context beneath it.

This study supports that clinical experience is not decisive for medical students to understand the sociocultural aspects of psychiatry. Preclinical students could, as they drawed on their personal experiences in the discussion, build up humanistic literacy from the course. What is critical in medical humanities education in psychiatry, hence, are not clinical experience nor medical knowledge, but cultivating students’ empathy, critical thinking skills and being about to recognize the sociocultural context beneath the clinical cases.

Our study, however, has its limitations. First, although we provided adequate guidelines for students in the open questions where they can share their experience freely, students still exhibited considerable variation in the quality of their responses. Some narratives were thoughtful, but others were of insufficient quality or off-topic, which may have affected the reliability of this study. Second, as a one-semester, cross-sectional exploratory qualitative study conducted at a medical school in Taiwan with an observational design without a control group, the current results cannot be generalized to other populations. It is expected that future research with similar course design and setting in different socio-cultural contexts could broaden our understanding of medical humanities education.

## Conclusion

This study demonstrated that scenario-based discussions led by a multidisciplinary team of facilitators can benefit students to contemplate psychiatric cases beyond biomedical aspects. Under this course design, medical students with limited clinical experience are able to reflect on the socio-philosophical aspects of psychiatry at different facets. The implementation of this pedagogical model during preclinical education can prevent interruption of clinical clerkship, narrowing the gap between medical humanities and clinical psychiatry education. Based on our findings, we also suggest that courses should emphasize the connection between the scenarios and students’ personal experiences or those of their friends and family, as these experiences enhance students’ empathetic understanding of others’ suffering.

## Data Availability

The datasets generated and/or analysed during the current study are not publicly available due to the privacy of the medical students but are available from the corresponding author on reasonable request.
